# Evaluation of Serum Albumin-Coated Bone Allograft for Bone Regeneration: A Seven-Year Follow-Up Study of 26 Cases

**DOI:** 10.3390/ijms24119232

**Published:** 2023-05-25

**Authors:** Kata K. Gyulay, Péter Karászi, Mátyás Rédei, Petra Sólymos, Károly Schandl, Zsombor Lacza, Dénes B. Horváthy

**Affiliations:** 1 Department of Interventional Radiology, Heart and Vascular Centre, Semmelweis University, 1122 Budapest, Hungary; gyulay.kata@semmelweis.hu (K.K.G.); redei.matyas@stud.semmelweis.hu (M.R.); solymos.petra@stud.semmelweis.hu (P.S.); 2 Department of Radiology, Medical Imaging Centre, Semmelweis University, 1083 Budapest, Hungary; 3 Department of Sports Surgery, Saint George University Teaching Hospital of County-Fejér, 8000 Székesfehérvár, Hungarykaroly.schandl@gmail.com (K.S.); 4 Department of Sports Physiology, University of Physical Education, 1123 Budapest, Hungary; 5 Translational Medicine Institute, Semmelweis University, 1085 Budapest, Hungary

**Keywords:** serum albumin, bone allograft, bone substitute, BoneAlbumin, bone–tendon–bone autograft, BTB, anterior cruciate ligament replacement, donor site morbidity, radiomorphology

## Abstract

We have previously reported that serum albumin-coated bone allograft (BoneAlbumin, BA) is an effective bone substitute. It improves bone regeneration at the patellar and tibial donor sites six months after harvesting bone-patellar tendon-bone (BPTB) autografts for primary anterior cruciate ligament reconstruction (ACLR). In the present study, we examined these donor sites seven years after implantation. The study group (N = 10) received BA-enhanced autologous cancellous bone at the tibial and BA alone at the patellar site. The control group (N = 16) received autologous cancellous bone at the tibial and blood clot at the patellar site. We evaluated subcortical density, cortical thickness, and bone defect volume via CT scans. At the patellar site, subcortical density was significantly higher in the BA group at both time points. There was no significant difference in cortical thickness between the two groups at either donor site. The control group’s bone defect significantly improved and reached the BA group’s values at both sites by year seven. Meanwhile, the bone defects in the BA group did not change significantly and were comparable to the six-month measurements. No complications were observed. There are two limitations in this study: The number of patients recruited is small, and the randomization of the patients could have improved the quality of the study as the control group patients were older compared to the study group patients. Our 7-year results seem to demonstrate that BA is a safe and effective bone substitute that supports faster regeneration of donor sites and results in good-quality bone tissue at the time of ACLR with BPTB autografts. However, studies with a larger number of patients are required to definitively confirm the preliminary results of our study.

## 1. Introduction

The best choice of graft type for anterior cruciate ligament reconstruction (ACLR) is highly debated among orthopedic professionals [1,2,3,4,5]. As evidenced by several scientific studies, reviews, and meta-analyses, excellent clinical outcomes can be achieved using autologous grafts such as hamstring and patellar tendon grafts [6]. Patellar tendon grafts in the form of bone–patellar tendon–bone (BTB) autografts are commonly used because they provide a stable fixation point and bone-to-bone healing at both ends of the graft with bone plugs, and they have high initial tensile strength and stiffness [7,8,9]. BTB autografts are a favored graft choice for professional athletes because they may promote rapid healing, restore the translation and rotation of the knee joint to close to normal, and result in higher return-to-sport rates than hamstring tendon autografts [6]. However, while offering comparable or superior benefits to other techniques, BTB autografts have also been reported to be associated with debilitating donor-site morbidities such as patellofemoral or anterior knee pain and residual limitation of joint motion [10,11]. These issues can limit the rehabilitation process of patients, particularly those at the professional athletic level [12,13]. As ACL injury and reconstruction incidence rates continue to increase and particularly affect young adults [14,15], there is a high demand for reliable techniques to mitigate these symptoms.

Research papers in the literature on the effect of donor-site healing on clinical symptoms after BTB autograft harvesting are controversial, mostly outdated, and mainly focus on left-open donor sites [16,17,18]. Previous data suggests that patellofemoral pain improves only approximately two years after the reconstructive surgery [13,19]; the reason behind this phenomenon is most likely the slow or inadequate bone regeneration at the donor sites. Long-term clinical outcomes and side effects related to donor site healing and corresponding radiological appearance after BTB autograft harvesting with modern techniques are lacking. Radiologic analysis of bone quality on computer tomography (CT) scans in a quantitative manner can include measuring the cortical thickness and the subcortical density (represents the transitional zone between compact cortex and pure trabecular bone) at a fixed depth for standardized values among patients [20,21,22]. 

Although autologous bone is considered the most effective treatment for addressing bone defects, the volume of bone material obtained from the tunnel drillings in this particular case is insufficient to fill the defects [23]. As a result, alternative bone substitutes must be explored. In this clinical use case, we consider biocompatibility, osteoconduction, osteoinduction, integration, availability, safety, and degradation to be the main considerations for choosing a bone graft material. Metallic and synthetic products are commonly utilized substitutes, with all options presenting different advantages and disadvantages, making graft selection dependent on the unique clinical situation they are used for [24,25,26,27]. Although metal substitutes are biocompatible with high mechanical strength and stiffness, they are unsuitable for donor-site bone substitution as they lack bioactivity, osteoconductivity, and osteoinductivity and do not integrate well with the surrounding tissue [24,25,26]. Synthetic and natural polymers, ceramics, and bioglasses are biocompatible and biodegradable, with versatile usage, as they offer high control over composition and reproducibility, and some ceramic and bioglass options even provide osteoconductive and osteoinductive properties [25,26,27]. Their disadvantages include low mechanical and fracture strength and unpredictable degradation rates [26]. Furthermore, there is prevailing evidence that specific ceramic grafts fail to decrease donor-site morbidity after ACL reconstruction with a BTB autograft [28]. Composites from the materials mentioned above combine their advantages and disadvantages and offer limited control over different properties and potential mismatched degradation rates [26]. Material science and tissue engineering advances continue to improve bone substitutes’ properties and overcome some of these limitations [24,29,30,31,32,33].

Bone substitutes from a biological origin are another category from which to choose bone grafts. Xenogenic bone poses the apparent risk of immunogenicity problems and disease transmission. In addition, patient acceptance regarding animal tissues is not universal [25]. Allogenic bone substitutes are commonly utilized due to their complete biocompatibility and strong osteoconductive properties [25,26,27,30,34]. However, their osteoinductive properties are not as favorable. To enhance this aspect, proteins, growth factors, or even stem cells may be utilized [35]. Despite their ability to achieve effective bone remodeling in experimental studies, these intricate and fully-equipped implants have limited practical application in less severe cases such as BTB surgery [10,36]. This highlights the urgent need for a cost-effective and less complex bone substitute with superior efficacy. Allogenic bone transplantation poses a small risk of disease transmission, even though there are only a few documented cases in the literature [25,37]. Therefore, the safety of these bone materials is ensured through careful donor selection, tissue quarantine, and tissue processing containing rigorous sterilization efforts to avoid even the slightest risk of transmission of infection [38,39].

Albumin in the human body is present as part of the blood circulation, lymphatic system, and extracellular and intracellular compartments. It accelerates the regeneration of various wounds and the healing of bone tissue. According to previous studies, albumin is successfully used as a biostatic structure, biomaterial coating, or highly biocompatible scaffold [32,33,40,41,42,43]. Tissue-engineered serum albumin hydrogels used as scaffolds offer a porous structure to achieve great fluid permeability and effective nutrition flow to provide better tissue regeneration [44]. Such scaffolds could be utilized in numerous clinical applications that require grafting materials that promote superior healing processes [45]. Furthermore, albumin is an active molecule; the mode of action is not yet fully clarified, but previous animal and in vitro studies have shown that after bone fractures, the local albumin concentration increases [44,46]. Even twelve months after albumin-coated bone allograft implantations, intensive osteoblast action can be observed [47]. Data in the literature suggest that albumin-coated allografts heal faster and create a higher detectable density in rats’ calvaria [42]. In vitro research has proven that serum albumin probably recruits endogenous stem cells and therefore stimulates bone regeneration locally, meaning that albumin has not only osteoconductive but also osteoinductive properties, making it a suitable bone substitution for optimal regeneration [33,40,43,48]. Numerous preceding studies support the use of bone albumin-coated allografts, which offer more effective bone regeneration at the donor site by stimulating mesenchymal stem cells to stick to the surfaces of the allograft and proliferate there [48,49]. Positive results of implanting bone albumin were observed in the case of non-union defects, and exalted long-term bone remodeling stimulant properties were detected [41,48]. BoneAlbumin, as an albumin-coated bone chip, takes advantage of these benefits of serum albumin while the allogenous bone acts as a scaffold. Our group’s previous research showed that patients who received BoneAlbumin allograft implants tend to have less donor site pain and have elevated, faster bone regeneration [50]. 

This research aimed to investigate the following inquiries: (1) Does the computed tomography (CT) morphology of donor sites seven years after the implantation of BoneAlbumin allografts indicate better bone quality compared to autogenous bone grafts or blood clots? (2) Is there a disparity in the volume of bone defects in the donor site between the control and the BoneAlbumin allograft groups? (3) Additionally, were there any long-term, radiologically detectable adverse effects associated with the BoneAlbumin allografts?

## 2. Results

### 2.1. Descriptive Statistics

**Figure 1:**
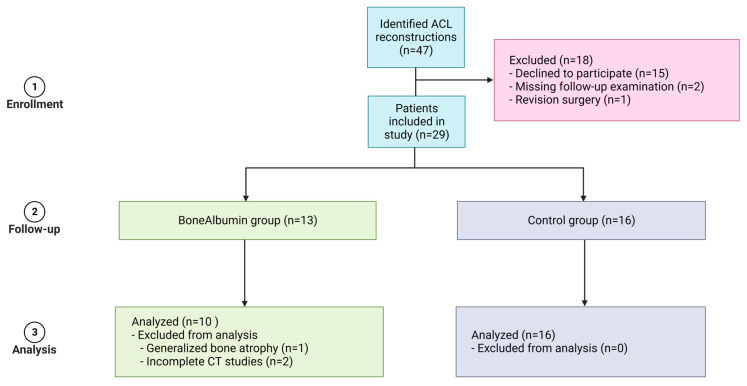
Flowchart of patient inclusions.

Initially, we were able to recruit 29 patients that met the inclusion criteria. After excluding three patients (one with generalized bone atrophy affecting both measured donor sites, two with missing imaging data), the study comprised ten patients (two female, eight male) in the BoneAlbumin (BA) group and sixteen patients (sixteen male) in the control group (Figure 1).

The median age was 28.5 years (IQR 27–42) in the BA group and 36 years (IQR 33–43.5) in the control group. Among the control group, six were professional athletes (37.5%), five were leisure athletes (31.25%), one had a sedentary lifestyle (6.25%), and four (25%) did not specify their activity level. The BA group included five professional athletes (50%), four leisure athletes (40%), and one person with a sedentary lifestyle (10%). Patients received treatment after 14.5 weeks (IQR 2–27.5) in the control group and after 14 weeks (IQR 6–16) in the BA group following the injury. The median follow-up period at the first CT after surgery was 199 days (IQR 195–207 days) in the BA and 198 days (IQR 194–206.5 days) in the control group. The long-term follow-up after reconstruction was at 82 months (IQR 82–83 months) in the BA group and 83 months (IQR 82–89 months) in the control group.

### 2.2. Bone Quality: Cortical Thickness

**Figure 2:**
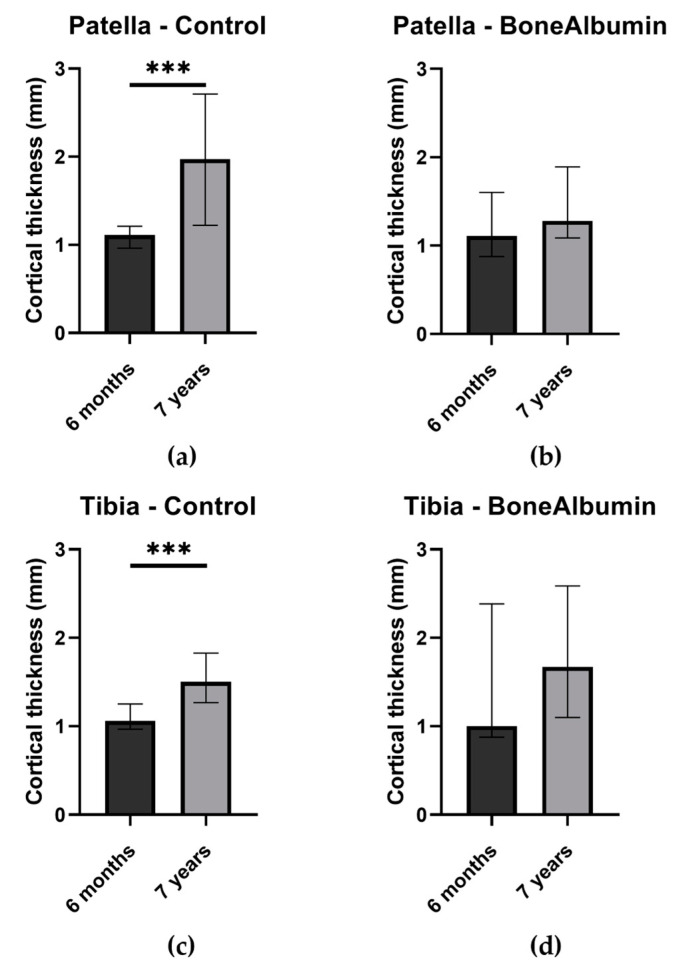
Cortical thickness (mm) by treatment groups and donor sites, measured at six months and seven years. Columns: median. Error bars: interquartile range. (**a**) Cortical thickness of the patellar site in the control group at both follow-up examinations. *** significant difference, *p* = 0.0006. (**b**) Cortical thickness of the patellar site in the BA group at both follow-up examinations. (**c**) Cortical thickness of the tibial site in the control group at both follow-up examinations. *** significant difference, *p* = 0.0009. (**d**) Cortical thickness of the tibial site in the BA group at both follow-up examinations.

When we analyzed the cortical thickness of both donor sites with the 6-month scans, we did not find any significant difference between the two groups. However, the BA group had higher values than the control group at both sites. On the 7-year scans, there was no significant difference between the two groups for either the patella or tibia, indicating proper long-term bone regeneration at both sites. Nevertheless, all values improved compared to the previous scans, and the control group’s improvement was significant at both donor sites (*p* = 0.0006 for the patella and *p* = 0.0009 for the tibia) (Figure 2). This suggests that the BA group had a faster bone regeneration rate than the control group, as the BA group’s measurements between the two time points were not significantly different.

At the tibial donor site, the BA groups’ measurements exhibited a wider distribution, which can be attributed to the presence of high cortical thickness values in specific individuals at both time points. Additionally, these patients displayed thicker cortical values at the patellar site, indicating that their cortical bone thickness values and bone regeneration may be influenced by individual factors outside the scope of our research.

### 2.3. Bone Quality: Subcortical Density

**Figure 3:**
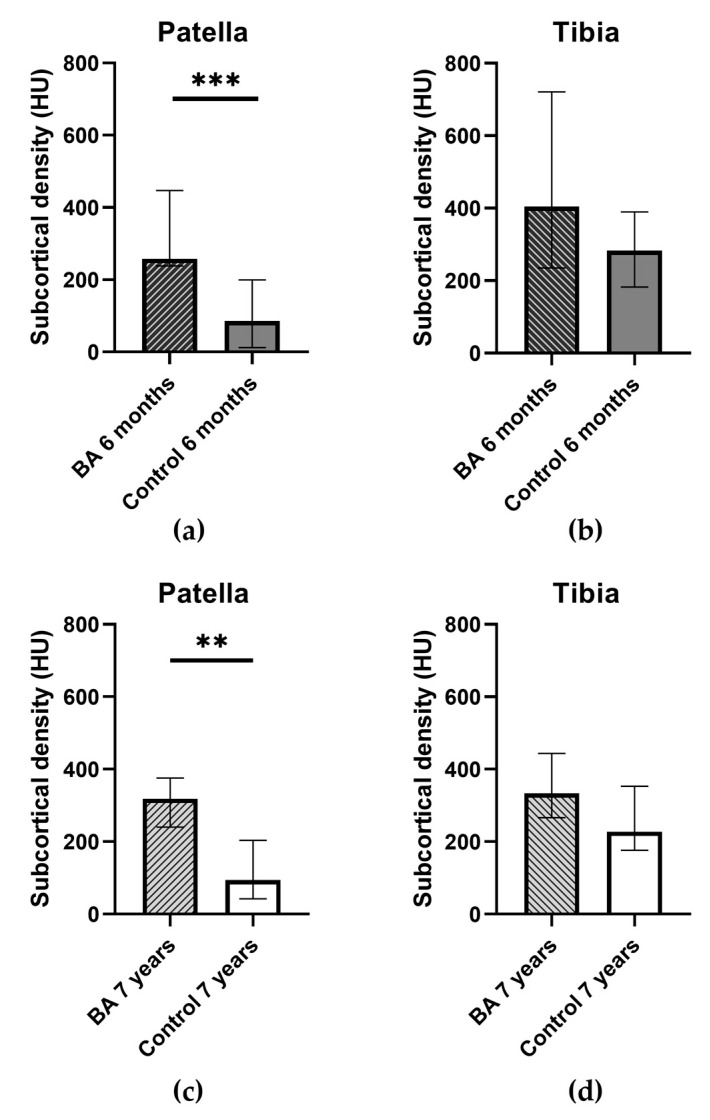
Subcortical densities at the measured sites. Columns: median. Error bars: interquartile range. (**a**) Patellar donor site by treatment group at 6 months. *** *p* = 0.0004 (**b**) Tibial donor site by treatment group at 6 months. No significant difference. (**c**) Patellar donor site by treatment group at seven years, ** *p* = 0.001. (**d**) Tibial donor site by treatment group at seven years. No significant difference.

In the patellar donor site, a significantly higher subcortical density was observed in the BA group than in the control group at both six months and seven years (*p* = 0.0004 and *p* = 0.0010, respectively) (Figure 3). Additionally, the control groups’ measurements at the patellar site showed a significantly lower value than their reference values at both time points (*p* < 0.0001 at six months, *p* = 0.0155 at seven years). The BA groups’ values did not indicate a significant difference from the reference values on either exam. Neither group’s values showed significant improvement between the 6-month and the 7-year scans (Table 1). All reference values showed no significant difference between each other.

Subcortical density at the tibial donor site did not differ significantly between the control and the BA groups or from any references at either six months or seven years. Both BA and control group values improved at this site over time, but not enough to mark a significant difference. By year seven at the tibial donor site, subcortical density in both groups was similar to their relevant reference values, indicating the active remodeling of the bone stock (Table 2). All reference values showed no significant difference between each other.

BoneAlbumin alone was used to compensate for the patellar bone defect in the BA group, and the donor site was filled with a blood clot in the control group. The blood clot-filling did not yield satisfactory bone quality, either in the short or long run. Subjectively, the BA group’s allografts resembled the subjects’ original bone matter more closely, whereas the control group’s donor sites exhibited more irregular, cystic, or compacted parts. Our observation suggests more optimal bone regeneration with BA allografting compared to using no bone graft for filling the donor site, and there was a comparable result between BA-enhanced autografting and autologous bone grafting in terms of subcortical density.

### 2.4. The Volume of Bone Defects

**Figure 4:**
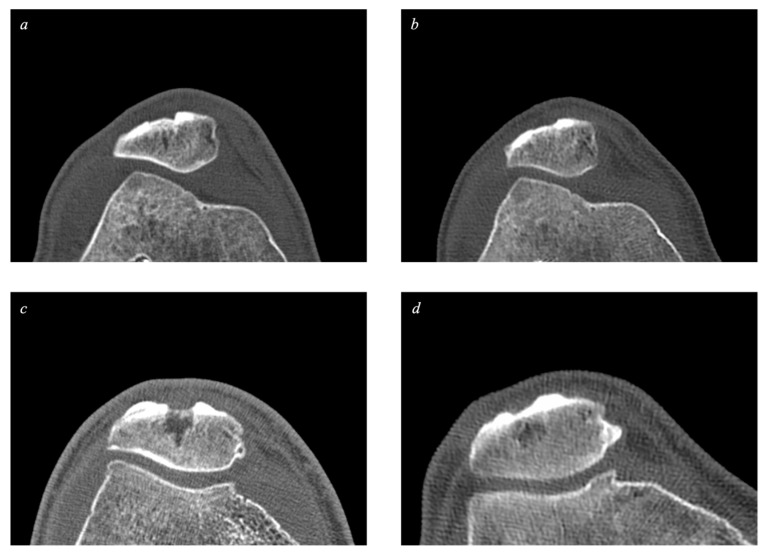
Patellar donor sites (bone window). The donor sites are located in the central part of the patella. Picture (**a**)*:* A patellar donor site from the BA group 6 months after surgery. Picture (**b**)*:* A patellar donor site from the same patient as picture (**a**), 7 years after surgery. Picture (**c**)*:* A patellar donor site from the control group 6 months after surgery. Picture (**d**)*:* A patellar donor site from the same patient as picture (**c**), 7 years after surgery.

**Figure 5:**
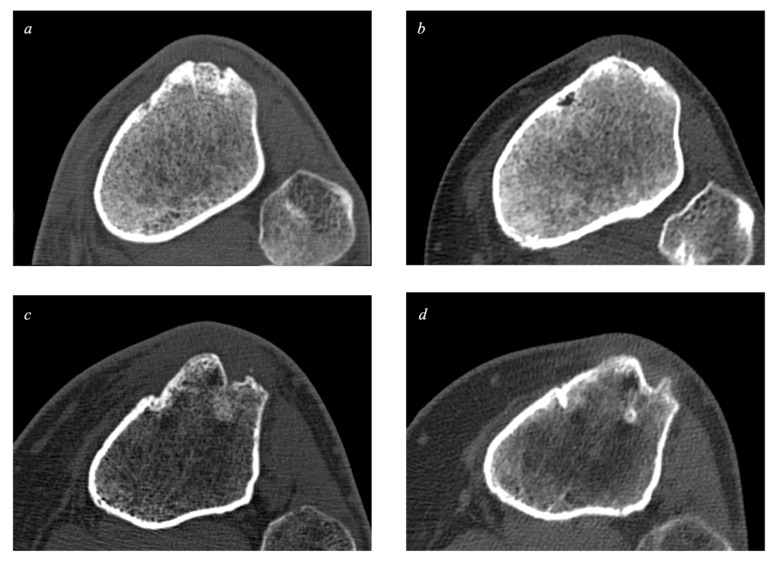
Tibial donor sites (bone window). Picture (**a**): A tibial donor site from the BA group 6 months after surgery. Picture (**b**): A tibial donor site from the same patient as picture (**a**), 7 years after surgery. Picture (**c**)*:* A tibial donor site from the control group 6 months after surgery. Picture (**d**): A tibial donor site from the same patient as picture (**c**), 7 years after surgery.

The bone defect was measured as the missing bone volume (mm^3^) at the donor sites. Figure 4 and Figure 5 demonstrate examples of the measured sites.

Examining the tibial site six months after surgery, it appears that the bone defect volume of the BA group is significantly lower compared to the control group (212.7 (IQR 136.8–327.7) and 494.1 (IQR: 347.2–571.6) mm^3^ respectively, *p* = 0.0006). This difference may be because the bone defect in the BA group was completely filled with autologous bone chips and BA, reforming the bone to near-normal contours, while only the available amount of autologous bone chips was added to the bone defect in the control group. At the same site, after seven years, the bone defect largely resolved in both groups and did not differ significantly. Examining the patella, both at six months and seven years, the BA group’s defect had a smaller median volume, but this was not significantly different from that of the control group.

**Figure 6:**
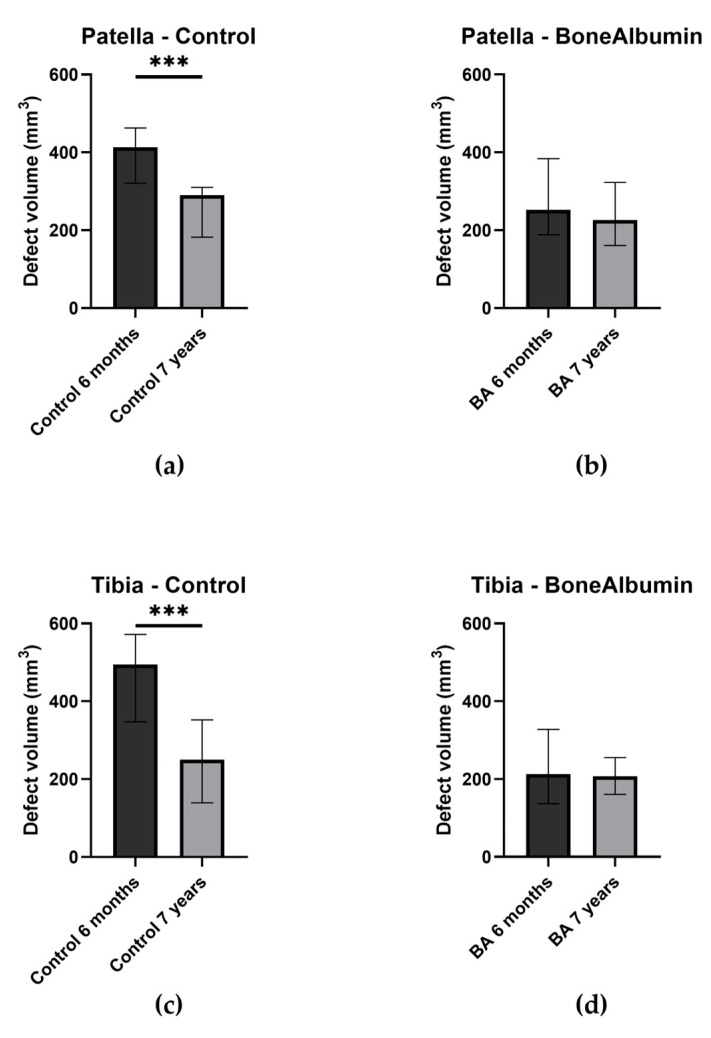
Bone defect volumes (mm^3^) of donor sites. Columns: median. Error bars: interquartile range. (**a**) Patellar site, control group at both follow-ups, *** significant difference, *p* = 0.0002. (**b**) Patellar site, BA group at both follow-ups; the difference is not significant. (**c**) Tibial site, control group at both follow-ups, *** significant difference, *p* < 0.0001. (**d**) Tibial site, BA group at both follow-ups; the difference is not significant.

However, if we assess the volume changes of the bone defect in the control group from the 6-month measures to the 7-year measures, it appears significantly reduced at both donor sites (*p* = 0.0002 for the patella and *p* < 0.0001 for the tibia) (Figure 6), while the BA group did not show significant changes over time at any donor sites.

### 2.5. Complications

We found no evidence of postoperative complications or adverse effects, such as sequestration, infection, or excessive ectopic bone formation, in any of the patients.

## 3. Discussion

For ACL reconstructions, BTB is a widely used solution for patients, even though postoperative donor site pain limits the use of this technique [1,6,11]. More rapid and better-quality bone tissue formation could be a backbone of donor site healing and, as such, result in improved clinical outcomes. Different studies have shown varying clinical outcomes regarding the use of bone grafting as a means to solve the problems associated with donor sites. Some reported that bone grafting the patellar defect did not alleviate kneeling complaints or patellofemoral problems, but they did not clarify the origin, type, or methods of the bone grafts used [13,51]. Others, including our team, proposed bone grafting as part of the solution to decrease donor-site problems [50,52,53]. There seems to be an agreement among professionals regarding donor site complaints improving with time [13,19], and some studies have shown that by combining bone grafting and special incision techniques to preserve the infrapatellar nerve, only 17% or fewer patients had anterior knee pain after two years [11,52], which is significantly less compared to the previously reported 32–43% [12,54,55]. However, as Hacken et al. highlight, there is no clear understanding of to what degree donor site morbidity influences the measurements of well-established and frequently used patient outcome tools used by the aforementioned studies. For this reason, they proposed a new scoring instrument to assess and differentiate donor site problems, but this tool has not been validated on an independent sample yet [11]. Radiological analysis with computer tomography is a reliable tool for a comparable and reproducible assessment of bone quality and quantity [20], but since BTB autografts’ donor site healing and corresponding radiological appearance after harvesting with modern techniques is lacking, interpretation of the results is challenging.

In our study, we use cortical thickness and subcortical density as a measure of bone quality. The regenerating bone at both six months and seven years was similar for both groups, and the BA group had slightly better values throughout. The change of cortical thickness over time in the two groups indicates different healing paces, as the BA group’s measurements reached similar-to-normal measures faster, while the improvement of the control groups’ measures stretched out in time, resulting in a significant difference between the two values at both sites.

Measuring the subcortical density on the tibial site revealed that no significant difference developed between the two groups at any time point, and seven-year values almost reached the respective reference subcortical densities. This fact indicates good long-term bone quality and donor site healing revealed in previous studies when using autografts. Evaluating the patellar sites, a significantly higher subcortical density was measured in the BA group compared to in the control group after both six months and seven years. This means that BoneAlbumin allograft is superior to a blood clot both short-term and long-term in terms of increasing bone quality at donor sites. Upon examining the BA groups’ donor site healing subjectively, it is evident that the use of albumin stimulated the integration of the graft and the regeneration of bone tissue, indicating that albumin fosters active bone healing.

Autograft amount for donor site grafting in the case of using BTB autografts for ACLR is variable and usually not enough to fill both donor sites, as the surgeon can only use the amount of corticocancellous bone from the drilling of the tunnels and leftover bone from the harvested autograft after fitting the graft into its place. In the treatment group in our study, the level of donor site grafting was always ‘complete’ because BA filling was used to top up the defects. In our previous study, leftover corticocancellous bone was sufficient only in 58% of the cases to fill one donor site only, leaving the other site (patellar in this case) completely open, resulting in slow tissue remodeling [50]. Six months after surgery, the bone defect of the BA group in the tibia remained significantly reduced compared to the control group. After seven years, the bone defect had largely been resolved in both groups, which also means a significant reduction of defect volume in the control group at both sites. This means that the BA group reached values similar to the smaller values of the 7-year scans sooner, indicating faster regeneration in regards to bone volume. The bone defect after BTB graft harvesting was approximately 10 × 10 × 20 mm in size and, as a consequence, did not count as a critical-sized bone defect [56,57]. Contrary to this classification’s suggestion of the high percentage of spontaneous healing of small and medium-sized defects, some studies suggest that in the specific case of a BTB graft’s donor site, there can be a persistent gap present, even thirteen years after the harvesting procedure [58]. Although in our study both patellar and tibial defects showed improvement in terms of defect volume over time, the aesthetic appearance of BTB autograft harvest sites may still play a role in young adults’ decision making when choosing a graft, as a distinct bony depression can be observed for years at left-open donor sites [28,58]. In the short term, the use of an allograft to fill the donor site can alleviate patients’ subjective cosmetic concerns following ACLR with a BTB autograft.

As ectopic bone formation in vivo is a prerequisite for categorizing a material as osteoinductive, a significant concern arises regarding its ability to stimulate excessive ectopic bone formation. The use of recombinant bone morphogenetic proteins (BMP) is an example of this concern, as recent literature suggests that the treatment may result in ectopic bone formation on the periphery of the implant, which is a side effect that has diminished the enthusiasm of clinicians [59,60,61]. It is important to note that the optimal dosage of BMP has not been well researched even though allogeneic bone substitutes may contain these proteins at levels that are naturally occurring [26]. Although our current study did not use specific growth factors, the fact that albumin triggers stem cell proliferation on the grafts’ surface raises questions about the possibility of excessive ectopic bone formation. Furthermore, implanted bone allografts can transmit infection, and the ability to avoid this kind of disease transmission is increasingly crucial for the successful use of biomaterials in vivo [26,39]. In this study, we found no evidence of ectopic bone formation, sequestration, or infection related to BoneAlbumin at any CT scans or follow-up examinations.

## 4. Materials and Methods

### 4.1. Study Protocol and Patients

We invited patients with available contact information to participate in the current study after undergoing ACL reconstruction with bone-patellar tendon-bone (BTB) autografts between 2012 and 2015 in a single center. Patients had simultaneous collateral ligament damage, rheumatological pathologies, and chondropathies higher than grade II. Outerbridge, pre-existing anterior knee pain, previous surgery on the affected knee, reoperation of the affected knee, and patients not presenting at any control appointments were excluded from the study. Most patients participated in high-level sports activities before their injury. After the first round of exclusions (1 patient due to revision surgery after reconstruction and 2 patients due to missing imaging studies), we were able to assess 29 patients between 23 and 56 years of age in a long-time follow-up study. Prior to the statistical analysis, we excluded one patient due to general bone atrophy presenting on their CT scans and two patients due to incomplete imaging studies. Our final analyzed study groups included ten patients in the BA and sixteen patients in the control group. The performed surgical protocols are detailed below in the ’surgical protocol’ subsection. All patients received detailed information about the risks and benefits of the procedure and the details of our study before granting informed consent to both and undergoing surgery. After the reconstruction, all patients underwent the same rehabilitation and follow-up protocol. The study was performed under the approval of the Semmelweis University Regional and Institutional Committee of Science and Research Ethics (SE RKEB 241/2020, date of approval: 15 December 2020). Patients were blinded toward the treatment group until after the 6-month scans.

### 4.2. BoneAlbumin-Serum Albumin-Coated Bone Chips

The West-Hungarian Regional Tissue Bank (Győr, Hungary) obtained bone fragments from cadavers or femoral heads during primary hip replacement surgery. These fragments were immediately processed using Urist’s method to create autolyzed and antigen-extracted allogenic bone. To preserve the bone grafts, they underwent freeze-drying and EtOH sterilization. After that, the bone grafts were immersed in a sterile solution of 10% human serum albumin for one minute, using low-salt-content Biotest human albumin infusion (Biotest Pharma GmbH, Dreieich, Germany). Following that, a second round of freeze-drying was conducted in sterile conditions with identical parameters, leading to the production of BoneAlbumin.

### 4.3. Surgical Protocol

All patients received ACL reconstruction using BTB autografts following our standard clinical protocol. The only difference between the control and the BA groups was the treatment of the donor sites. The BTB autografts were obtained by harvesting the middle third of the patellar tendon, including approximately 10 mm × 10 mm × 20 mm bone blocks from the patella and tibia. The remaining ACL was removed through a medial arthrotomy. The tibial bone tunnel was prepared measuring 9–10 mm in diameter, while the femoral tunnel was placed on the medial surface of the lateral femoral condyle and had a diameter of 10–11 mm. The tibial part of the BTB autograft was secured in the femoral tunnel with an interference screw. After the graft was tensioned, the patellar bone block was stabilized in the tibial tunnel with two titanium Kirschner wires.

Autologous corticocancellous bone derived from the tunnel drillings was used to repair the tibial donor site in the control group, while the patellar donor site was filled with a blood clot. In the treatment group, BA was manually mixed with equal volumes of autologous corticocancellous bone to fill the tibial donor site, and BA alone was used to fill the patellar defect. Approximately 3 cm^3^ of allograft chips were required to sufficiently fill either donor site.

### 4.4. Computed Tomography

Patients underwent computed tomography (CT) of the knee at both six months and seven years post-surgery using Philips Brilliance 64 and Philips Incisive 128 (both from Koninklijke Philips N.V., Amsterdam, The Netherlands). The scans utilized 120 KV and 200–300 mA to obtain axial slices with a thickness of 0.9 mm and an increment of 0.45 mm. Various parameters were measured, including cortical thickness, subcortical density, and bone defect volume at the patellar and tibial donor sites, as well as the subcortical density of adjacent normal bone for reference. Cortical thickness was calculated as the average of five measurements taken from different axial slices in the donor site area. Subcortical density in Hounsfield Units (HU) was measured at the donor sites by manually placing a circular region of interest (ROI) measuring 20 mm^2^ approximately 2 mm below the cortex. To establish a reference value, the same technique was used to measure the subcortical density of normal bone next to the donor sites. The bone defect volume was measured by hand on each axial slice using the pencil tool and then combined to form a 3D model of the bone defect. Ectopic bone formation, sequestration, and other complications were also evaluated on donor sites. The group assignment was kept undisclosed to the person evaluating the images. Philips IntelliSpace Portal Client v12.1.6.11044 (Koninklijke Philips N.V., Amsterdam, The Netherlands) was used for all measurements and 3D reconstructions. Analysis and measurements were conducted in an appropriate room through a proper Barco Nio Color 2MP LED MDNC-2221, 21” (Barco N.V., Kortrijk, Belgium).

### 4.5. Statistical Analysis 

All metric values are reported as the median and interquartile range (IQR). Statistical analysis was performed using the Mann–Whitney U test for independent samples and the Wilcoxon signed-rank or the Friedman test for dependent samples in GraphPad Prism software (v8.0.1; San Diego, CA, USA); *p* values < 0.05 were considered significant. Sample size estimation was performed beforehand with TIBCO Statistica Software (v14.0.1.25, Cloud Software Group, Inc., Fort Lauderdale, FL, USA) based on relevant data from similar studies. The sample size was calculated to be at least seven for each group, with a probability of less than 5% for alpha (type I error) and a power of 95%.

### 4.6. Limitations

There are a few limitations in this study. The number of patients recruited in this study is small. Besides that, the randomization of the patients could have improved the quality of the study, as the control group patients were older compared to the study group patients. Despite these limitations, this report has reached a few statistically significant conclusions. Evaluation by a blinded investigator and a control group are the strengths of this report.

## 5. Conclusions

Our study’s conclusions are (1) BoneAlbumin allograft implantation at the donor sites results in good quality regenerated bone tissue, similar to the patients’ original values. BoneAlbumin offers a faster healing rate and is obviously superior to a blood clot in both the quality and quantity of the formed bone tissue at six months as well as at seven years. It also offers faster bone regeneration than plain corticocancellous autografts. (2) The BA group’s defect volume stayed at a constant low level, and the control group’s defect volumes reduced from the 6-month measurements significantly at both sites, resulting in no significant difference in the bone defects’ volume between the control and the BA group at seven years. (3) We found no radiologically detectable adverse effects related to BoneAlbumin.

## Figures and Tables

**Table 1:** Median-, upper-, and lower-quartile subcortical density values at the patellar site in Hounsfield units. Both groups had their own reference values (ref.) at both time points, measured in the normal bone mass. Number of measured sites are presented in the first row. Significant differences between the BA and the control groups are highlighted in Figure 3.

	BA Ref. 6 Months	BA 6 Months	BA Ref. 7 Years	BA 7 Years	Control Ref. 6 Months	Control 6 Months	Control Ref. 7 Years	Control 7 Years
Valid number	10	10	10	10	16	16	16	16
25th percentile	142.3	238.3	206.3	240.5	222.3	12	180.3	42.5
Median	275	258.5	264.5	318.5	247	85.5	244	94.5
75th percentile	295.3	446.8	282.5	375.8	275.2	199.5	316	203.8

**Table 2:** Median-, upper-, and lower-quartile subcortical density values at the tibial site in Hounsfield units (HU). Number of measured sites are presented in the first row. Both groups had their own reference values (ref.) at both time points, measured in the normal bone mass.

	BA Ref. 6 Months	BA 6 Months	BA Ref. 7 Years	BA 7 Years	Control Ref. 6 Months	Control 6 Months	Control Ref. 7 Years	Control 7 Years
Valid number	10	10	10	10	16	16	16	16
25th percentile	227	234.8	181.5	266	214.8	182.3	222.5	176.5
Median	336.5	404	330.5	333.5	306	283.3	312	227.5
75th percentile	405.5	721	377.5	443.8	382.8	389.8	374.3	352.8

## Data Availability

The data presented in this study are available on request from the corresponding author.
